# Upregulation of the HOXA10-AS1 LncRNA in Gastric Cancer: An Expression and Bioinformatics Analysis

**DOI:** 10.30699/ijp.2025.2046969.3385

**Published:** 2025-11-11

**Authors:** Farideh Ghanbari Mardasi, Sharareh Eskandarieh, Reza Taslimi, Mohsen Eghtedari, Zeinab Jamali, Masoomeh Safaei, Majid Kabuli, Reza Shirkoohi

**Affiliations:** 1 Department of Medical Genetics, School of Medicine, Tehran University of Medical Sciences, Tehran, Iran; 2 Cancer Biology Research Center, Cancer Institute of Iran, Imam Khomeini Hospital Complex, Tehran University of Medical Sciences, Tehran, Iran; 3 Multiple Sclerosis Research Center, Neuroscience Institute, Tehran University of Medical Sciences, Tehran, Iran; 4 Department of Internal Medicine, Division of Gastroenterology, Tehran University of Medical Sciences, Tehran, Iran; 5 Department of Biotechnology, Faculty of Biotechnology, Amol University of Special Modern Technologies (AUSMT), Amol, Iran; 6 Department of Pathology, School of Medicine, Cancer Institute, Tehran University of Medical Sciences, Tehran, Iran

**Keywords:** Gastric cancer, HOXA10-AS, lncRNAs

## Abstract

**Background & Objective:**

Gastric cancer (GC) is a lethal disease with poor prognosis. Long non-coding RNAs (lncRNAs) involved in the development of cancer through changes in their expression levels. In present study, we aimed to evaluate HOXA10-AS gene expression and its potential as a biomarker in GC.

**Methods:**

In this study 60 subjects (30 gastric carcinoma tissues and 30 adjacent non-carcinoma tissues) were examined. The expression level of the HOXA10-AS gene was evaluated using quantitative PCR. Furthermore, clinicopathological characteristicswere taken into consideration. Diagnostic value of the HOXA10-AS was examined by ROC curve analysis. Bioinformatics analysis was performed using different databases.

**Results:**

Expression of HOXA10-AS was significantly upregulated in GC tumoral tissues. Roc curve analysis revealed that the diagnostic power of HOXA10-AS was high (AUC = 0.64) in the tumor compared to the normal GC tissues.

**Conclusion:**

Our findings show that HOXA10-AS expression level is higher in the GC tumor compared to the adjacent non-carcinoma tissues and can act as a strong diagnostic biomarker in GC patients.

## Introduction

Gastric cancer (GC) is the fifth most commonly diagnosed cancer worldwide (1–2). Approximately 40% of patients with GC are diagnosed at the metastatic stage, and only about 5% of these patients survive beyond five years (3). Based on genetic characteristics, GC is recognized as a heterogeneous disease comprising diverse subtypes, each with distinct molecular features and clinical behaviors (4). Although recent advances have improved the early detection of GC, prognosis and treatment outcomes for patients with advanced-stage disease remain poor. Therefore, identifying novel molecular targets and elucidating the roles and mechanisms of dysregulated molecules are critical for understanding GC pathogenesis and improving therapeutic strategies (5–6).

Long noncoding RNAs (lncRNAs) are transcripts longer than 200 nucleotides that do not encode proteins (7). Previous studies have shown that lncRNAs play significant roles in regulating gene transcription, post-transcriptional modifications, epigenetic processes, and protein translation (8). They are involved in various biological functions, including cell differentiation, tumorigenesis, and chromatin remodeling (9). Moreover, lncRNAs have been implicated in promoting cellular proliferation, tumor invasion, and metastasis across multiple cancer types (10–12). Several studies have reported that lncRNA dysregulation correlates with tumor characteristics and prognosis in patients with GC. Specific lncRNAs have been proposed as potential diagnostic or prognostic biomarkers for GC (13). Given their diverse functions in cancer progression, lncRNAs represent a promising source of novel cancer biomarkers and therapeutic targets.

In the present study, we focused on the long noncoding RNA HOXA10 antisense RNA1 (HOXA10-AS) to evaluate its expression levels in GC tissues and adjacent non-cancerous tissues (ANCTs) in an Iranian population. HOXA10-AS is transcribed antisense to the HOXA10 gene. According to the LncRNADisease v2.0 database (http://www.rnanut.net/lncrnadisease/), HOXA10-AS is implicated in the pathogenesis of several malignancies, including gastric cancer, nasopharyngeal carcinoma, pancreatic neoplasms, oral cancer, glioma, and leukemia. Notably, HOXA10-AS expression is upregulated in several cancers and functions as an oncogene in glioma and oral cancer (14–15). This study aims to explore the potential of HOXA10-AS as a diagnostic or prognostic biomarker for GC.

## Materials and Methods

### Bioinformatic analysis

LncRNADisease v3.0 database (http://www.rnanut.net/lncrnadisease/) was used to evaluate whether HOXA10-AS expression was involved in GC. Then, the web tool GEPIA (http://gepia.cancer-pku.cn/) was utilized to obtain and evaluate the Cancer Genome Atlas data and to assess HOXA10-AS1 expression. Also, ENCORI database (https://rnasysu.com/encori/) was used to assess HOXA10-AS interaction with other RNAs.

### Sample collection

In this study, 60 carcinoma tissues (n = 30) and corresponding ANCTs (n = 30) were gathered from the tumor bank, Imam Khomeini Hospital, Tehran, Iran. Tissues were collected during regular surgery prior to any adjuvant chemoradiotherapy. Tissue samples were stored at −70 °C until molecular evaluations. The clinicopathologic features such as age, gender, tumor size, grade, stage, invasion to lymph nodes, peritoneal, and vascular were provided by the tumor bank. Patients were on average 65.4 years old (range: 44 to 82). The study protocol was approved by the Ethical Committee of Imam Khomeini Hospital (IR.TUMS.IKHC.REC.1402.307), and informed consent was obtained from all the subjects.

### DNA extraction and quantitative real-time PCR (q-RT-PCR)

The Trizol reagent was used to extract total RNA from the specimens. An agarose gel electrophoresis 1/5% was used to evaluate the quality of the extracted RNA. After evaluating the purity of RNA using NanoDrop (NanoDrop 2000, Thermo Scientific), A260/A280 ratios ranging from 1.8 to 2 were considered for further investigation. The RNA underwent DNase enzyme treatment without the presence of RNase in order to eliminate any DNA contamination. Then, RNA was transformed to complementary DNA (cDNA) by the Karmania Pars Gene cDNA Synthesis Kit.

Primers for amplification of genes were designed by online Primer-BLAST software ((https://www.ncbi.nlm.nih.gov/tools/primer - blast/) and summarized in Table 1. The quantitative real-time PCR (q-RT-PCR) was carried out by RealQ Plus 2x Master Mix Green no Rox (Ampliqon, Denmark) in a Light-Cycler96 Roche thermocycler Real Time PCR system (Applied Biosystems, USA). All experiments were carried out in duplicate, and the specificity of the PCR product was verified by melt curve analysis from 60 to 95 °C. Beta-actin was used as an internal control gene based on previous study (16). Data analysis of q-RT-PCR was calculated using the -△Ct method.

### Statistical analysis

Data analysis was performed by GraphPad Prism 8.4.3 and IBM SPSS Statistics v.26 software. Relative expression of HOXA10-AS gene was compared between the two groups (gastric carcinoma tumors and ANCTs) by the paired sample *t-*test. Mann-Whitney and one-way ANOVA assessments (Kruskal-Wallis) were applied for evaluation of the relationship between the expressions of HOXA10-AS gene and clinicopathological characteristics of patients. A P-value of < 0.05 was regarded as significant. The receiver operating characteristic (ROC) curve was used to determine the potential of this gene for GC diagnosis.

**Table 1:** The primers and PCR product length

Gene name	Primer sequence	Primer length (bp)	Product length (bp)
HOXA10-AS	F: GCTGCGGAGAAAGACACGA R: TCTCGACAGCACGACACTG	19 19	165
B-Actin	F: GACATTAAGGAGAAGCTGTG R:GAGTTGAAGGTAGTTTCGTG	20 20	212

## Results

### Bioinformatics Analysis and Expression of HOXA10-AS

The LncRNADisease v3.0 database confirmed the involvement of HOXA10-AS in gastric cancer (GC). Mutations in this transcript were associated with altered HOXA10-AS expression. Data retrieved from the ENCORI database indicated interactions between HOXA10-AS and multiple RNA-binding proteins (RBPs), as summarized in Table 2.

**Table 2:** Data extracted from the ENCORI database on interaction of HOXA10-AS with RNA binding proteins.

Gene Name	Gene Type	RBP
HOXA10-AS	lncRNA	CHTOP
		CPSF6
		CSTF2T
		DHX36
		ELAVL1
		LIN28B
		PTBP1
		RBM15
		RBM15B
		RBMX
		SCAF4
		SCAF8
		SOX2
		TARDBP
		U2AF1
		YTHDF1

**Fig 1:**
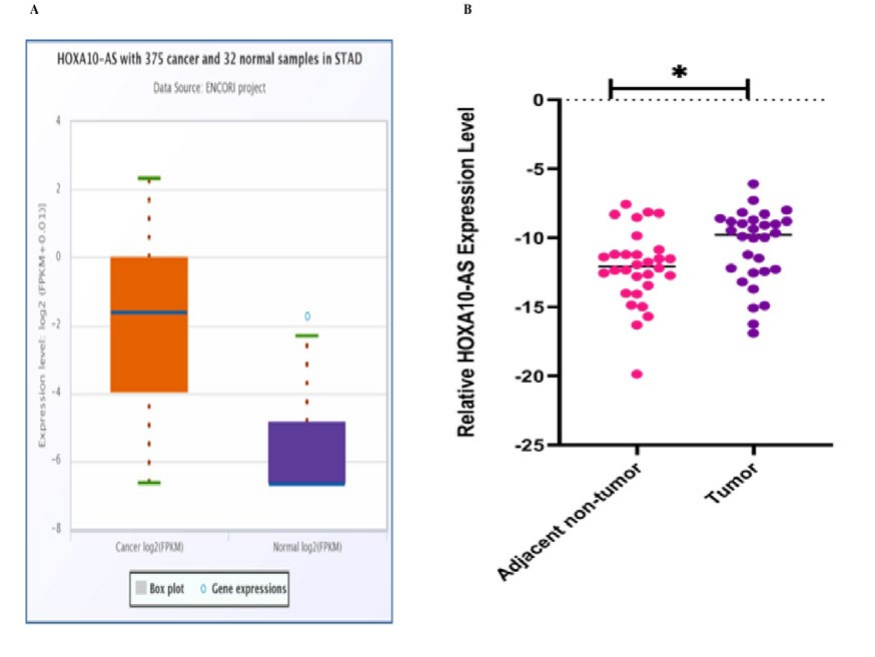
A) TCGA database revealed increased HOXA10-AS expression in STAD. (B) qRT-PCR analysis of HOXA10-AS in GC tissues (n = 30) vs. ANCTs (n = 30).

Transcriptomic data from The Cancer Genome Atlas (TCGA), comprising 408 GC tissues and 211 normal tissues, were analyzed using the GEPIA web tool. The analysis showed a significant upregulation of HOXA10-AS in GC tissues compared to normal counterparts (P < 0.05) (Figure 1A). Furthermore, ENCORI database analysis identified interactions between HOXA10-AS and approximately 16 different RNAs.

To experimentally validate HOXA10-AS expression, quantitative reverse transcription PCR (qRT-PCR) was performed on 30 paired samples of GC tumors and adjacent non-cancerous tissues (ANCTs). The results demonstrated significantly higher expression of HOXA10-AS in tumor tissues compared to ANCTs (P = 0.01) (Figure 1B).

### Correlation Between HOXA10-AS Expression and Clinicopathological Characteristics

To assess whether elevated HOXA10-AS expression is associated with clinical features, we compared expression levels across different clinicopathological parameters (Table 3). The analysis revealed no statistically significant associations between HOXA10-AS expression and any of the evaluated clinicopathological characteristics (P > 0.05).

**Fig. 2:**
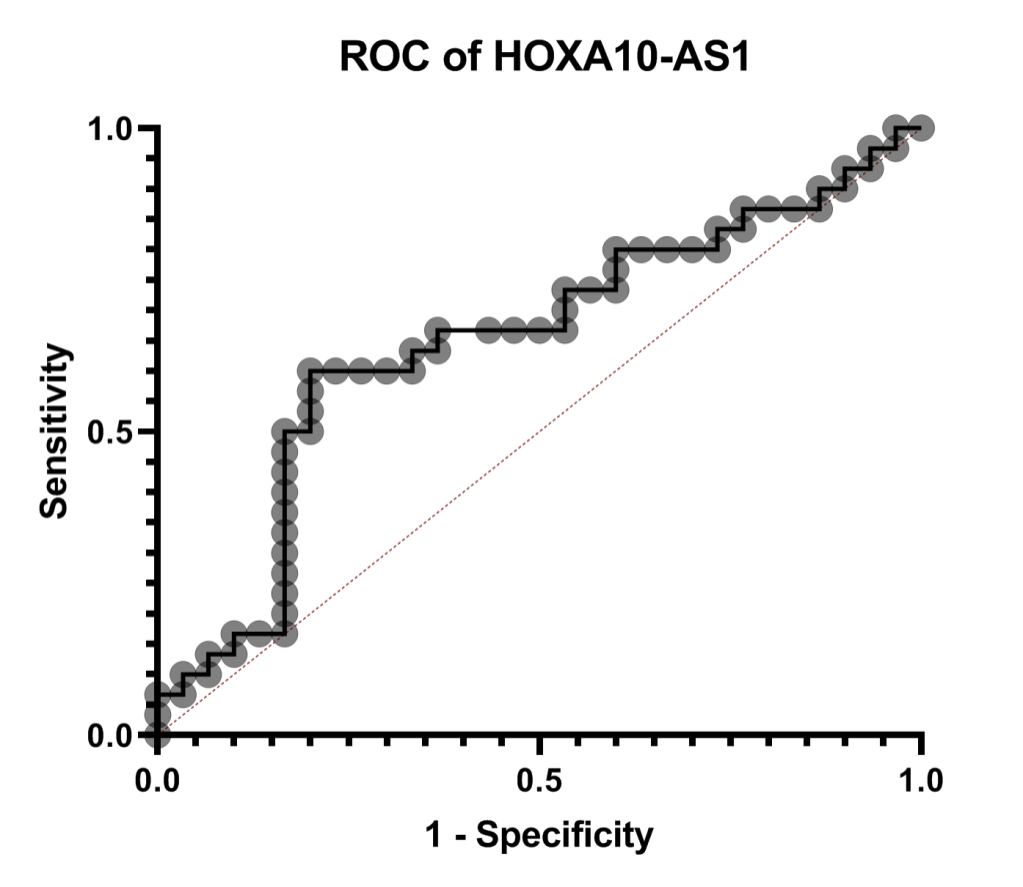
ROC curve of HOXA10-ASin gastric cancer patients.

### Evaluation of the Diagnostic Potential of HOXA10-AS

Receiver operating characteristic (ROC) curve analysis was conducted to evaluate the diagnostic performance of HOXA10-AS expression in distinguishing GC tissues from ANCTs. The area under the curve (AUC) was 0.64 (Figure 2), indicating moderate discriminatory ability. These findings suggest that HOXA10-AS may serve as a potential biomarker for GC detection.

**Table 3:** Correlation between the expression of lncRNA- HOXA10-AS GC patients' Clinicopathological characteristics (n = 30).

Characteristics	Subclass	N (%)	Mean	SEM	P value
Age (Years, mean (range))	<65	17 (56.67%)	10.57	0.5616	0.82
	65 or >65	13(43.33%)	10.81	0.91	
Gender (number (%))	Male	24 (80%)	10.55	0.55	0.65
	Female	6 (20%)	11.15	1.18	
Site of primary tumor (number (%))	Cardia	10 (33.33%)	9.85	0.59	0.33
	Body	12 (40%)	10.61	0.86	
	Antrum	8 (26.67%)	11.78	1.10	
Tumor diameter	<4 cm	10 (33.33%)	9.496	0.66	0.15
	4 or <4 cm	20 (66.67%)	11.26	0.64	
Histologic grade (number (%))	2	17 (56.67%)	10.65	0.66	0.97
	3	10 (33.33%)	10.59	0.9	
	4	3 (10%)	11.02	1.95	
Stage	II	6	9.20	0.7	0.18
	III	17	10.64	0.73	
	IV	7	11.99	0.84	
Lymph node invasion (number (%))	yes	20 (66.67%)	10.16	0.53	0.2
	no	10 (33.33%)	11.70	1.0	
Vascular invasion (number (%))	yes	20 (66.67%)	10.70	0.60	0.77
	no	10 (33.33%)	10.61	0.91	
Peritoneal invasion (number (%))	yes	16 (53.33%)	10.87	0.65	0.67
	no	14(46.67%)	10.44	0.78	

## Discussion

Cancer is a complex, multifactorial disease influenced by both genetic and environmental factors (12). The identification of reliable diagnostic, prognostic, and therapeutic targets remains a major focus of ongoing cancer research. An ideal biomarker should reflect normal or pathological biological processes and be readily detectable in blood, body fluids, or tissues. Key features of effective biomarkers include utility in early detection and diagnosis, prognostication, prediction of treatment response, and monitoring of disease progression or recurrence (17).

It is now well established that more than 98% of the human genome is transcribed into non-coding RNAs (ncRNAs), while protein-coding genes represent less than 2% of the genome (18). The functions of many ncRNAs, particularly long non-coding RNAs (lncRNAs), remain poorly understood. However, growing evidence highlights the important roles of lncRNAs in tumorigenesis and their potential as therapeutic targets (19). Despite increasing research, the role of lncRNAs in gastric cancer (GC) remains underexplored.

In this study, we investigated the clinical relevance of the lncRNA HOXA10-AS in an Iranian cohort of patients with GC. In silico analysis indicated that HOXA10-AS may function as a competing endogenous RNA (ceRNA), regulating mRNA expression through RNA–RNA interactions. Data from the ENCORI database revealed that HOXA10-AS interacts with various RNA-binding proteins (RBPs) (Table 1), suggesting its involvement in the epigenetic regulation of gene expression. Previous studies have shown that HOXA10-AS is dysregulated in several malignancies, including oral squamous cell carcinoma (OSCC) (14), glioma (15), and lung cancer (21). For instance, HOXA10-AS overexpression in OSCC correlated with poor prognosis (14), and in glioma, elevated HOXA10-AS expression was associated with higher tumor grade. Its knockdown was shown to suppress cancer cell proliferation (15). In lung cancer, HOXA10-AS upregulation was also linked to increased cell proliferation and unfavorable prognosis (21).

Our findings demonstrated that HOXA10-AS expression was significantly upregulated in GC tissues compared to adjacent non-cancerous tissues, supporting its potential oncogenic role in gastric tumorigenesis. Although we found no significant associations between HOXA10-AS expression and clinicopathological features such as tumor stage, grade, or metastasis, this lncRNA may still serve as a candidate biomarker for early GC detection. Larger studies are needed to evaluate its prognostic and therapeutic relevance.

Previous research has reported associations between lncRNA expression and tumor stage or grade (20). For example, Yan et al. found that HOXA10-AS expression was significantly higher in high-grade OSCC (P < 0.001) (14), and Dong et al. reported similar findings in gliomas, where HOXA10-AS was more strongly expressed in high-grade (III and IV) than low-grade (I and II) tumors (15). In contrast, our study did not find any statistically significant associations between HOXA10-AS expression and clinicopathological parameters such as age, sex, tumor size, lymph node involvement, or vascular/peritoneal metastasis in GC patients. The limited sample size may partly explain these negative findings and restrict the statistical power to detect such associations.

Furthermore, receiver operating characteristic (ROC) curve analysis showed that HOXA10-AS could distinguish GC tissues from adjacent non-tumor tissues with an AUC of 0.64. This moderate diagnostic accuracy suggests that HOXA10-AS may serve as a supportive biomarker in identifying GC, although further validation in larger, multicenter cohorts is warranted.

Overall, the dysregulated expression of HOXA10-AS in gastric cancer highlights its potential role in GC pathogenesis and underscores the need for further investigation into its underlying molecular mechanisms and clinical utility.

## Conclusion

In summary, lncRNAs are increasingly recognized as key regulators of various physiological and pathological processes through their interactions with other molecular components. Our study reveals that lncRNA HOXA10-AS is significantly upregulated in gastric cancer tissues, suggesting its potential as a diagnostic biomarker for early detection of GC. These findings offer promising insights into the functional role of HOXA10-AS in GC tumorigenesis and malignant progression. Future research is needed to elucidate the molecular pathways governed by HOXA10-AS and to explore additional molecules within its regulatory network that may serve as therapeutic targets or prognostic indicators.

## Data Availability

All data generated during the study are included in this article. Further enquiries can be directed at the corresponding author.
